# The Effect of Fluid Therapy Before Spinal Anesthesia on Prevention of Headache After Cesarean Section: A Clinical Trial

**DOI:** 10.7759/cureus.11772

**Published:** 2020-11-29

**Authors:** Kiana Babaei, Mostafa Khaleghipoor, Seyedeh Masoumeh Saadati, Alireza Ghodsi, Nastran Sadeghi, Najmeh Nikoo

**Affiliations:** 1 Anesthesia, Neyshabur University of Medical Sciences, Neyshabur, IRN; 2 Operation Room, Neyshabur University of Medical Sciences, Neyshabur, IRN; 3 Statistics, Hakim Sabzevari University, Sabzevar, IRN; 4 Students Research Committee, Neyshabur University of Medical Sciences, Neyshabur, IRN

**Keywords:** fluid therapy, spinal anesthesia, headache, cesarean, clinical trial, pdph

## Abstract

Background: Despite numerous studies on postdural puncture headache (PDPH) and the factors affecting it, issues such as prevention methods and techniques that are associated with a lower prevalence of this complication are still under discussion and research.

The aim of this study was to evaluate the effect of increasing fluid therapy of patients before surgery on the incidence of postoperative headache.

Methods: This single-blind clinical trial study was performed on 60 patients undergoing elective surgery with spinal anesthesia based on the inclusion criteria in 2017 in Neyshabur. After obtaining the consent of the patients, the participants were randomly divided into two groups of intervention (A) and test (B) (30 people in each group). Data were created by self-checklist and visual analog scale (VAS) pain measurement criteria were recorded by phone during 4, 7, 24, 48, 72 hours, and 7 days after surgery.

Results: In the study, the average headache increased up to 72 hours after surgery in the experimental group and in the intervention group up to 48 hours after surgery and then decreased.

Conclusion: The results of our study generally showed that fluid therapy did not reduce headache, but showed decreasing trend of headache. According to the research results, more research is needed on the causes of headache after spinal anesthesia.

## Introduction

One of the most common methods for cesarean section is spinal anesthesia and performing this technique as the best method of anesthesia to prevent maternal and fetal complications is increasing [1-2]. At the same time, using this method has side effects such as hypotension, shortness of breath, nausea and vomiting, and headache. Postdural puncture headache (PDPH), one of the most important delayed complications following spinal and epidural anesthesia, was first reported in the late 19th century [3]. Due to the loss of cerebrospinal fluid (CSF), the volume and pressure are reduced, leading to the brain sinking into the cerebral cavity and stretching the meninges and other pain-sensitive structures and on the other hand, in order to maintain the cranial volume, the blood vessels inside the skull dilate, both of which can cause the patient a headache [4-5]. Miller and Pardo, also reported headaches following spinal anesthesia as the most common complaint, which could be due to the low outflow of CSF and pressure on the nerves in this area [6]. PDPH is a characteristic of the anterior and occipital region (back of the head) and may be accompanied by nausea and vomiting, auditory and visual symptoms, diffuse neck pain, and stiff neck. This type of headache is related to the patient's positioning so that it is intensified in the vertical position (sitting) and is relieved when resting [7]. It is more common in pregnant women due to age and gender [8]. Many treatments have been suggested to relieve PDPH, such as immobilization, fluid intake (VANZA), opioids, caffeine, somatriptan, dexamethasone, hydrocortisone, gabapentin [9], and also ondansetron [10]. If these treatments are unsuccessful, epidural blood transfusions are given [11]. Occurrence of PDPH varies from 1% to 40%, according to needle gage, needle orientation, operator skill level, and presence of risk factors such as patient's age or history of PDPH [12]. Oral hydration is another common treatment for PDPH, but there is no evidence that hydration is beneficial for patients with fluid retention problems [13]. Oral or IV fluids (three liters or more daily) compensate for the CSF deficiency, but its effectiveness in reducing headaches is yet to be determined. Despite numerous studies on PDPH and the factors affecting it, issues such as prevention methods and techniques that are associated with lower prevalence of this complication are still under discussion and research. To date, no study has investigated the effect of increasing preoperative serum therapy on the incidence of headache. Also, due to the occurrence of psychological problems and increased hospital costs in patients due to this complication and the lack of definitive prevention methods for such headaches, we decided to assess whether administration of supplementary fluid after lumbar puncture can prevent the onset of PDPH in people undergoing lumbar puncture for diagnostic or therapeutic purposes.

## Materials and methods

This single-blind clinical trial was performed on 60 patients undergoing elective surgery with spinal anesthesia (95% CI, 80% β) and based on other studies [14-16]. Inclusion criteria in this study were: age over 18 years, American Society of Anesthesiologists classification (ASA)II, personal consent of the patient, elective surgery, no history of cardiovascular disease, no history of migraine and seizures, no use of drugs and cigarettes or no drug and alcohol addiction problems, no coagulation problems, no history of pre-eclampsia and eclampsia, no history of spinal surgery, no previous history of spinal anesthesia, and no symptoms of increased intracranial pressure. Exclusion criteria were: noncooperation of the patient, failure to perform spinal, more than three times of dural perforation, excessive bleeding during the surgery, hypotension of more than 20% of the initial level not controlled with ephedrine and atropine, and incomplete spinal anesthesia that required anesthetics.

After obtaining informed consent and recording demographic information through a questionnaire, the samples were divided into two groups of intervention (A) and test (B) (30 in each group) using randomized block design. According to hospital routine, patients received an average of only 100 cc of normal saline before spinal. In the test group, after entering the operating room, patients routinely received serum (100 cc normal saline) while in the intervention group subjects received 500 cc of 0.9% normal saline in addition to serum, before the spinal anesthesia. On a normal diet, the minimum water intake is estimated at 500 mL/day (assuming there are no increased losses) [17]. Patients were put in the supine position before spinal surgery and were not subjected to any pre-medications. At this time, after connecting the monitoring, noninvasive systolic and diastolic blood pressure, heart rate, arterial blood oxygen saturation, electrocardiography, which was the standard monitoring in this study, were measured and recorded as baseline values in the relevant checklist. Then patients in both groups were placed in a sitting position and the spinal was performed by an anesthesiologist with 12 mg of bupivacaine 0.5% with Quinck needle number 27 (K-3point - length 90 mm- made by Dr J Chiana) at the vertebral space L4-L5 or L3-L4. After confirmation of the needle insertion in the subarachnoid space, the syringe content was slowly injected into the intrathecal space. To counteract the side effects of the medications, a reduction of more than 20% in the initial amount of systolic blood pressure was considered as hypotension requiring treatment and a reduction in heart rate to less than 45 beats per minute was considered as bradycardia in need of treatment which were treated with 10 mg IV ephedrine and 0.5 mg IV atropine, respectively. In case of nausea or vomiting, this condition was treated with 4 mg IV ondansetron. Subjects in need of a higher dose than expected or another medication were excluded. The degree of nausea and vomiting and medications received during the operation were recorded in the checklist. Patients were examined in the postoperative period in terms of severity of headache and back pain using the visual criterion of pain measurement, VAS. VAS is valid for registration of pain intensity in headache and nonheadache patients. Test-retest evaluation, effect sizes, and Cohen's delta values ere < 0.029 with < 1.5% change from test to retest (p < 0.01). Correlation coefficients were > 0.95 [18]. Scores are based on self-reported measures of symptoms that are recorded with a single handwritten mark placed at one point along the length of a 10-cm line that represents a continuum between the two ends of the scale-“no pain” on the left end (0 cm) of the scale and the “worst pain” on the right end of the scale (10 cm). VAS is scored from 0 to 10 as follows (0 = without pain, 1-3 =mild pain, 4-7 = moderate pain, and 8-10 = severe pain). Assessments were conducted at 4, 7, 24, 48, 72 hours and also 7 days after surgery through phone-calls while the questioner was blinded regarding the type of intervention.

Statistical analysis

The Shapiro-Wilk test was used to test the normality of quantitative variables. To compare the mean of the two independent groups, t-test was used if the data of both groups were normal, and otherwise Mann-Whitney test was utilized. Two groups were compared in terms of employment, gender, and the level of education using the Fisher's exact test. ANOVA test with repeated measures was used to compare the mean score of headache and back pain between the two intervention and test groups during the treatment period. The significance level of all statistical tests was considered as 0.05.

## Results

**Figure 1:**
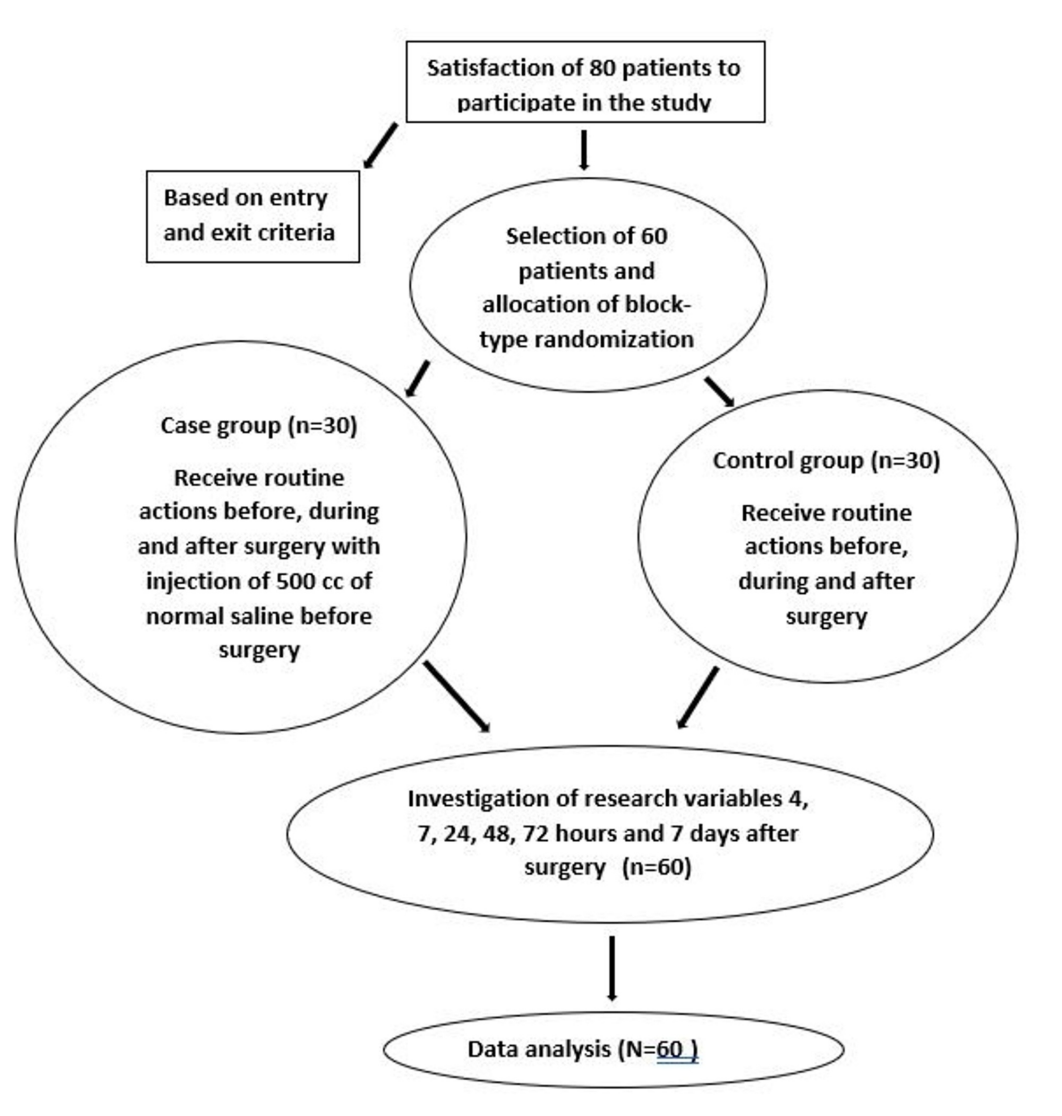
Clinical trial chart.

In this study, 60 patients (30 in the intervention and control groups) were studied and one was excluded from the sample due to failure to complete the questionnaire. The mean age of patients in the intervention group was 29.8365 ± 6.654 and in the control group it was 30.83 ± 7.938 years. The mean body mass index (BMI) in the intervention and the control groups was 29.093 ± 4.861 and 27.602 ± 3.911, respectively. The values of other demographic variables are shown in Table 1. t-test for independent samples showed that there was no significant difference in terms of age, BMI, length of operation, and heart rate between the intervention and case groups (p > 0.05). Mann-Whitney test also did not show any significant difference in terms of systolic blood pressure and oxygen saturation percentage in the two intervention and case groups (p > 0.05), but indicated significantly different levels of diastolic blood pressure between the two groups (p < 0.05). The difference regarding employment and education level between the two groups was not statistically significant based on Fisher's exact test (p > 0.05). Clinical trial chart is shown in Figure 1. 

**Table 1:** Information on demographic variables.

Diastolic pressure	Systolic pressure	Length of operation	Body mass index (BMI)
78.27 ± 3.43	122.60 ± 4.75	42.16 ± 3.85	29.09 ± 4.86
73.24 ± 2.16	124.14 ± 2.91	33.62 ± 2.04	27.60 ±.3.91

**Figure 2:**
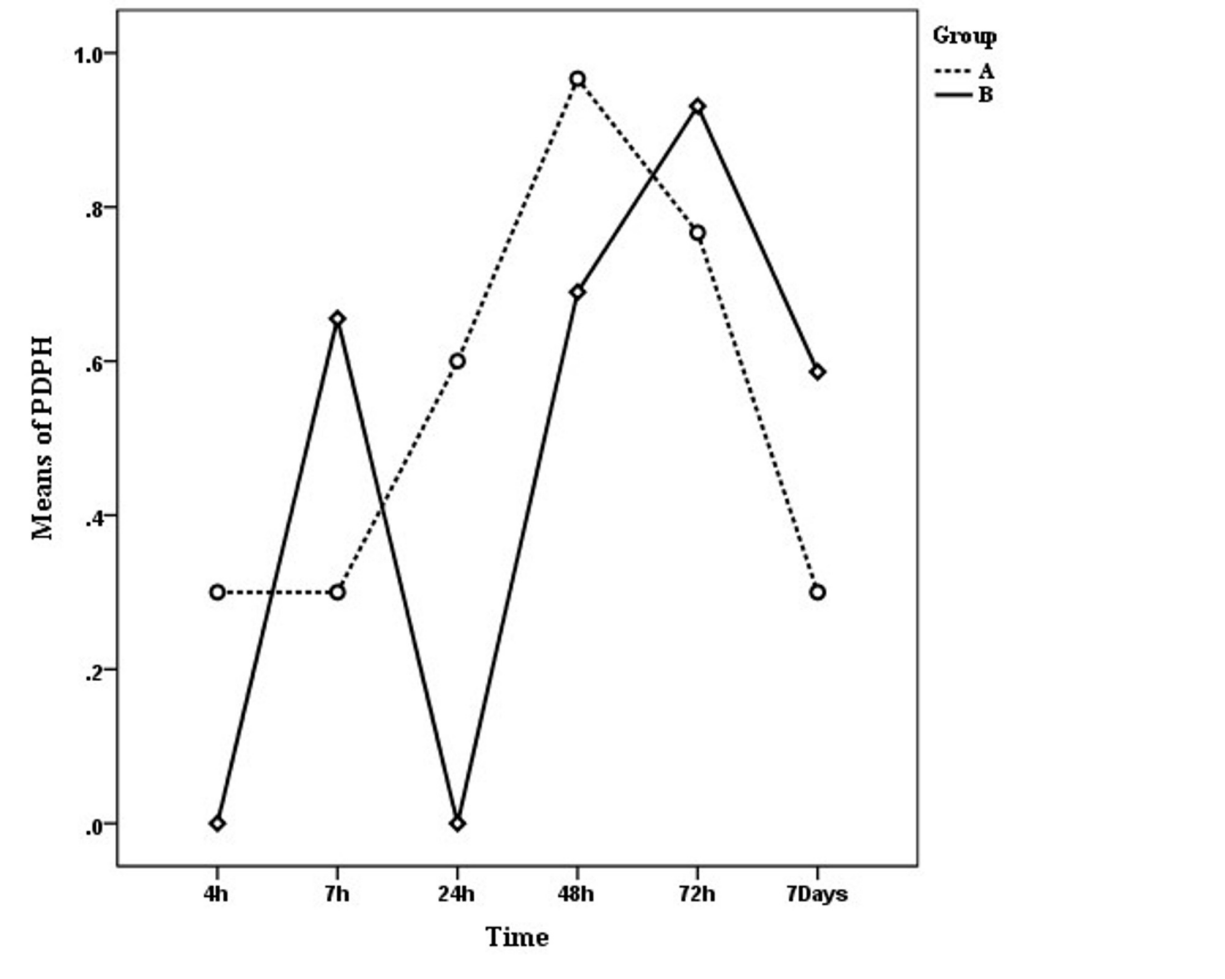
Mean headache at different times in two groups of intervention (A) and test (B).

Figure 2 and Table 2 show the mean score of headaches at 4, 7, 24, 48, and 72 hours and also 7 days after surgery in the intervention (A) and test (B) groups. As it can be seen by the graph, the mean score of headache increased until 72 and 48 hours after surgery in the experimental group and in the intervention group, respectively, and after this time, these scores were reduced. However, according to the Greenhouse-Geisser statistics in the ANOVA test using repeated measures, there were no significant differences in terms of headache level at different times (p > 0.05) (the power of this test is 0.52). While the mean headache score in the intervention group in the first 48 hours was higher and then less than the test group, ANOVA with repeated measures also did not show a significant difference in this regard (p > 0.05) (the power of this test is 0.06). Fisher's exact test also confirmed the ANOVA test results and showed that there was no relationship between headache score and preoperative serum therapy (p > 0.05). In addition, Student’s t-test did not indicate any significant difference in terms of the headache scores of the two groups in any of the measured times (p > 0.05). On the other hand, ANOVA with repeated measures and Fisher's exact test showed that the degree of headache depends on the job type (p < 0.05). Based on these tests, students and employees experienced more pain in the first 4 and 7 hours after the operation than others. Spearman correlation test also showed that the headache rate was independent of variables such as age, BMI, length of operation, heart rate, blood pressure, oxygen saturation percentage, and frequency of dural ruptures (try) (p > 0.05). The mean score of headaches among those with try numbers equal to two and three was less than those for whom the number of tries was equal to one, but ANOVA with repeated measurements showed that this difference was not significant (p > 0.05).

**Table 2:** Descriptive statistics of headache.

Time	Group		95% CI of differences		p-Value	Power of the test
	A	B	Lower	Upper		
4 h	0.30 ± 1.21	0.00 ± 0.00	-0.15	0.75	0.18	-
7 h	0.30 ± 1.21	0.66 ± 1.99	-1.21	0.5	0.41	0.23
24 h	0.60 ± 1.77	0.00 ± 0.00	-0.06	1.26	0.07	-
48 h	0.97 ± 2.13	0.69 ± 2.27	-087	1.42	0.63	0.08
72 h	0.77 ± 1.91	0.93 ± 2.37	-1.28	0.96	0.77	0.06
7 days	0.30 ± 0.99	0.59 ± 2.23	-1.18	0.61	0.52	0.10

**Figure 3:**
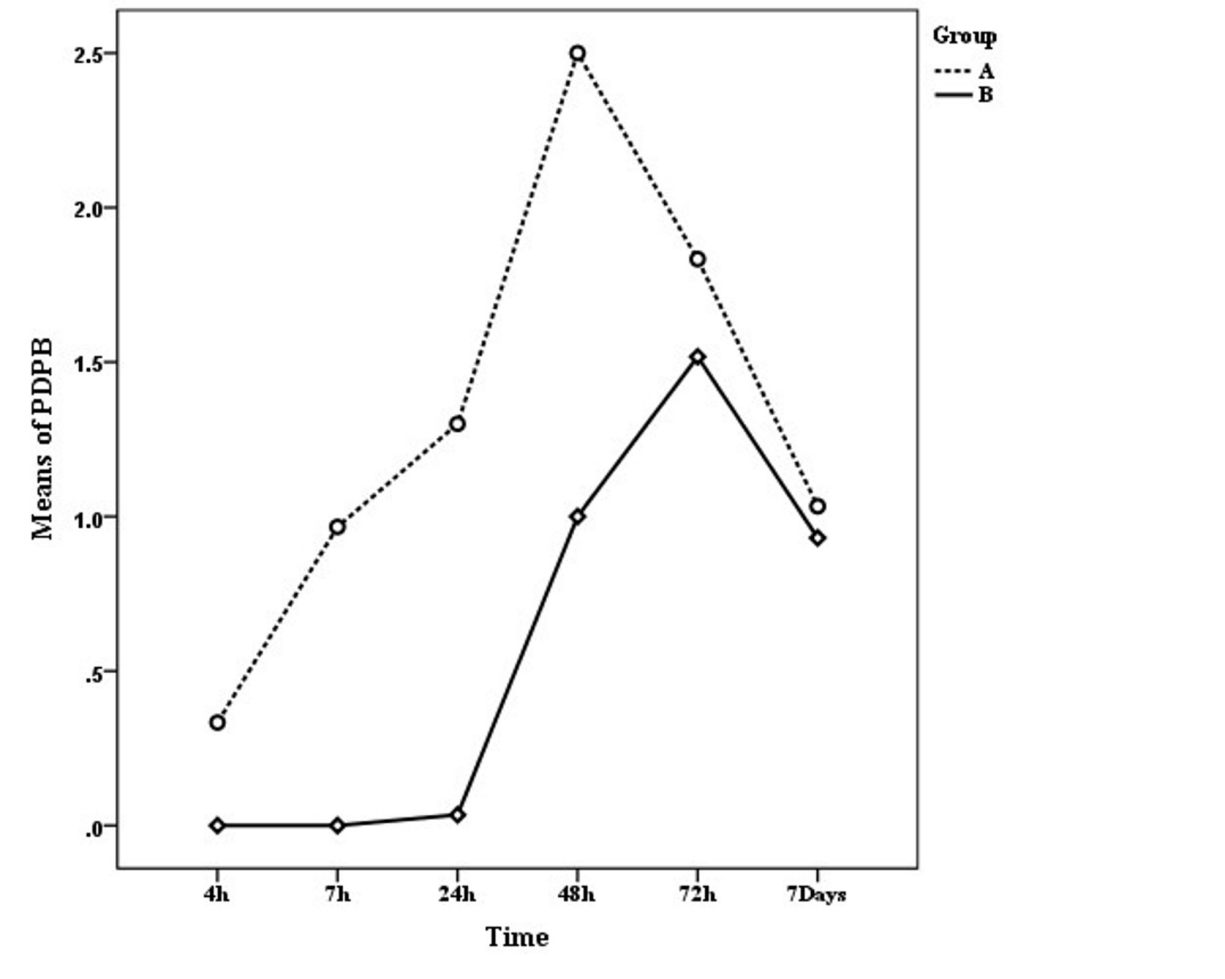
Mean low back pain at different times in two groups of intervention (A) and test (B).

Regarding other spinal complications in participating patients, as shown in Figure 3 and Table 3, the average level of low back pain increased until 72 and 48 hours after surgery in the experimental group and in the intervention group, respectively, and after that, a reduction occurred in these variables. Greenhouse-Geisser in ANOVA test with repeated measures also showed a significant difference in the level of low back pain at different times (p < 0.05) (the power of this test is 0.95). ANOVA with repeated measures also demonstrated that the score of low back pain after surgery in the control group was constantly lower than the group that received serum therapy before surgery (p < 0.05) (the power of this test is 0.52). Fisher's exact test did not find any significant relationship between nausea, vomiting, and chills with preoperative serum therapy (p > 0.05).

**Table 3:** Descriptive statistics of back pain.

Time	Group		95% CI of differences		p-Value	Power of the test
	A	B	Lower	Upper		
4 h	0.33 ± 1.83	0.00 ± 0.00	-0.35	1.02	0.33	-
7 h	0.97 ± 2.73	0.00 ± 0.00	-0.06	1.99	0.06	-
24 h	1.30 ± 2.60	0.03 ± 0.19	0.30	2.24	0.01^*^	0.54
48 h	2.50 ± 3.60	1 ± 2.60	-0.14	3.14	0.07	0.44
72 h	1.83 ± 3.25	1.52 ± 2.95	-1.30	1.93	0.70	0.07
7 days	1.03 ± 2.48	0.93 ± 2.05	-1.09	1.29	0.86	0.05

## Discussion

Postdural puncture headache is the most important delayed complication of spinal anesthesia [19]. Current treatments are not definitive, so it seems necessary to explore new treatment methods and ways to prevent headaches. The aim of this study was to determine the effect of preoperative serum therapy on the prevention of headache after spinal anesthesia. Findings of the study did not show any statistically significant relationship in terms of the rate and severity of postoperative headache in the intervention and control groups. However, the onset of pain reduction in the intervention group was faster than in the experimental group (48 hours postoperatively in the intervention group and 72 hours after surgery in the experimental group). In the experimental group, after 48 hours, the headache level started to decrease which was less than in the control group, but this difference was not significant. Also, fluctuations in headache severity in the experimental group were higher as compared to the intervention group. In the intervention group, the level of headache did not increase up to seven hours after the operation and was constant, while in the experimental group, an increase was observed up to seven hours after the operation and was higher than the intervention group. This constancy in the intervention group can be indicative of intravascular volume compensation (due to serum therapy) up to seven hours after surgery. Because in case of volume deficiency, the cerebral arteries dilate to compensate for this reduction in volume, which in turn leads to a headache [4-5]. In general, at seven and 72 hours and seven days, the rate of headache in the intervention group was less than the control, but this difference was not significant. There was no relationship between the type and volume of IV fluid therapy during and after the surgery between the two groups. Based on their observations, some researchers believe that the patient's hydration can affect the incidence rate of PDPH [20]. In other words, while volume preload decreases the incidence and degree of sympathetic blockage associated with spinal anesthesia, it does not appear to affect the prevalence of headache [21]. In summary, normal hydration must be maintained. Excessive hydration dose not reduce the headache, but dehydration worsens the symptoms. Gosch et al. [22] considered administration of sufficient fluids, use of smaller needles and special needles as prevention methods for headache after spinal anesthesia to reduce the patient's fear. In this study, the same needle size was considered to be used by the anesthesiologist. Considering that no research has been done in this field so far, it may be necessary to further investigate this issue with a larger sample size and to explore its degree and severity after one week while taking into account the culture and customs of the studied region in order to obtain accurate information with more generalizability. However, this research is significant due to its new and innovative inference.

In this study, also the types of variables based on surgical procedures such as duration of surgery, history of headache or back pain, history of spinal anesthesia, hemodynamic symptoms of patients before, during and after surgery, frequency of tries to insert the needle into the dorsum and other complications associated with spinal anesthesia such as low back pain, chills, nausea, and vomiting were compared along with the temporal factors. The results showed that the headache rate was independent of variables such as age, BMI, length of operation, heart rate, blood pressure, oxygen saturation percentage, and number of tries (p > 0.05). In a study by Haghighi et al. [23], no significant relationship was observed between gender, age, needle number, and headache. However, in this study, the used needle number and the anesthesiologist were the same throughout the sampling period. In Rahimi et al. study [24] and Kempen et al. study [25], no significant relationship was observed between headache and hemodynamic oscillations of the patient, same as in our study. In Nazemi's study [26], a significant relationship was observed between BMI and headache, which was contrary to our findings. On the other hand, the mean score of headache among those with try numbers equal to two and three was less than those for whom the number of tries was equal to one, but ANOVA with repeated measurements showed that this difference was not significant. Regarding other spinal complications in participating patients, ANOVA with repeated measures showed that fluid therapy significantly reduced low back pain, which was consistent with the study conducted by Haghighi et al. [23]. On the other hand, ANOVA with repeated measures and Fisher's exact test showed that the degree of headache depends on the job type (p < 0.05). Based on these tests, students and employees experienced more pain in the first four and seven hours after the operation than others which is in line with studies carried out by Wu et al. [27] and Haghighi et al. [23]. Some studies have shown an inverse relationship between PDPH and aging. Patients between the ages of 20 and 40 are more sensitive, with the lowest incidence occurring after the fifth decade of life [28-29]. In summary, many studies have suggested factors influencing the prevalence of PDPH including age, gender, pregnancy, previous history of post-spinal headache, needle size, needle tip shape, arc orientation to dorsal fibers, number of needle insertion attempts, MEDIN technique (midline) versus PARAMEDIN technique (lateral), type of local anesthetic solution, and clinical experience of the anesthesiologist [30]. In our study, these control factors were controlled and only the effect of fluid therapy on the rate of headache was investigated.

## Conclusions

Postdural puncture headache can increase the workload of physicians, patients' hospital stay, and treatment costs. Diagnosis of PDPH should be made when other causes of headache are ruled out. There are significant variables which are indicated in the incidence of PDPH, including age, gender, number of attempts, type of needle (design) and its size, history of previous PDPH or chronic headache, and anesthesiologist’s clinical experience which were controlled in our study and only the effect of fluid therapy was evaluated. The results of our study generally showed that fluid therapy did not reduce the rate of headache and back pain, but the decreasing trend of headache and back pain occurred more rapidly in the intervention group than in the control group (48 hours postoperatively in the intervention group and 72 hours postoperatively in the experimental group). According to the results of the study, more research is needed on the factors causing headache after spinal anesthesia, such as the type and dosage of anesthetics, patient’s experience of pain, quality of postoperative education to prevent headache, and adherence to treatment by the patient. The limitations of this study include small sample size, lack of measurement of headache in both groups at baseline, headache examination after one month, and lack of control for underlying variables such as patient’s mobility (absolute rest days), lifestyle, and nutrition.
